# An ethno-medicinal study of medicinal plants used for the treatment of diabetes

**DOI:** 10.15171/jnp.2016.08

**Published:** 2015-12-16

**Authors:** Babak Baharvand-Ahmadi, Mahmoud Bahmani, Pegah Tajeddini, Nasrollah Naghdi, Mahmoud Rafieian-Kopaei

**Affiliations:** ^1^Madani Heart Hospital, Lorestan University of Medical Sciences, Khorramabad, Iran; ^2^Food and Beverages Safety Research Center, Urmia University of Medical Sciences, Urmia, Iran; ^3^Medical Plants Research Center, Shahrekord University of Medical Sciences, Shahrekord, Iran; ^4^Clinical Microbiology Research Center, Ilam University of Medical Sciences, Ilam, Iran

**Keywords:** Medicinal plants, Diabetes mellitus, Hypertension

## Abstract

*Background:* Diabetes is the greatest public health problem and is considered as the silent epidemic of the 21st century. In Iran, there are approximately 1.5 million diabetic patients. Before the discovery of insulin, medicinal plants were widely used for the treatment of diabetes in Iran.

*Objectives:* This study aimed to determine the indigenous plants used for the treatment of diabetes in Shiraz, southwest of Iran.

*Materials and Methods:* Semi-structured direct interviews were conducted with 25 herbalists to identify medicinal plants used to treat diabetes. Questionnaires were included herbalist personal information, plant local name, growth season, plant parts used, preparation methods, and traditional therapies.

*Results:* The interview data indicated that, 24 medicinal plants from 19 families are used for the treatment of diabetes in Shiraz. The families with most antidiabetic species were Compositae (13%), Rosaceae (13%) and Cucurbitaceae (8%). The most frequently used plant parts were fruits (38%) and the most common preparation method was decoction (62%). For 45% of reported plants, pharmaceutical studies approved antidiabetic effects in animal or humane model of diabetes. Results of this study showed that the plants recommended by Shirazian herbalists have potential antidiabetic effects.

*Conclusions:* It is suggested that the ingredients of indigenous plants be studied to determine therapeutic effects and mechanism of action. If they were safe and effective, they can be refined and processed to produce natural drugs.

**Implication for health policy/practice/research/medical education:**

Diabetes is considered as the silent epidemic problem of the 21st century. The use of medicinal plants is particularly valuable in treatment of cardiovascular and metabolic disorders especially for diabetes. This is a good ethno-botanical study in which the authors present valuable medicinal plants effective on diabetes in Shiraz, south of Iran.

## 1. Background

Diabetes is the greatest public health problem which is considered as the silent epidemic of the 21st century (1). Diabetes is a chronic disease that gradually affects many different organs of the body. The disease progresses gradually and its symptoms occur several years after the onset of disease. During this period, serious and irreversible complications arise (2).

Diabetes is a treatable disease, however, when it is not under control, the risk of some other diseases, especially cardiovascular disorders will increase. It happens because most of patients with diabetes also have other conditions such as hypertension, hyperlipidemia, obesity and lower physical activities which greatly contribute to the pathogenesis of cardiovascular diseases. In this regard, it is estimated that in patients with both diabetes and hypertension, the risk of cardiovascular diseases doubles. Diabetic patients often have hyperlipidemia that is mostly associated with premature coronary heart disease. Insulin resistance is also increased in hyperlipidemia. In addition, obesity is associated with insulin resistance and is an important risk factor for heart diseases. More importantly, physical inactivity is another risk factor cardiovascular disease and insulin resistance. Exercising and losing weight have been proved to reduce blood pressure, prevent type 2 diabetes, and reduce the risk of cardiovascular diseases (3). Diabetes type 2 is the most common form of diabetes accounts for about 90%-95% of all diabetes cases. The prevalence of type 2 diabetes has increased dramatically in recent decades worldwide. Type 2 diabetes is a silent disease and many patients are unaware of their situation. As the disease progresses and its complications such as kidney and eye damages appear they become aware of their condition (4-7).

Increased public information about the symptoms, risk factors, prevention strategies and treatment of the disease have been associated with increased level of public health, reducing morbidity and mortality, and improving quality of life for persons (8,9). In Iran, the number of people with diabetes is estimated to be 1.5 million (10). The World Health Organization (WHO) has estimated that the total numbers of patients with diabetes in the world will rise from 135 million in 1995 to 300 million in 2025 (11). Chronic nature of the disease and its complications lead to financial burden and reduction of the quality of life in patients and their family (12).

Despite the incurable nature of metastatic cancer, it is believed that a majority of patients can gain adequate control of the disease through self-care activities. Before the discovery of insulin in 1921, diabetes controlling was referred to the prevention of early death from the disease. Todays, diabetes controlling is not only to keep blood glucose, lipid and pressure levels within a normal range, but also to prevent related complication and improve patient satisfaction and quality of life (13,14).

People interested in herbal medicines, believe that the use of herbal medicines dates back to the period when there were no modern medicine and no information about the cellular and molecular function of body (15). Although medicinal plants have a long history of use in the treatment of diseases however, their acceptance and application in modern medicine need time (16). However, this alternative medicine is still attractive for people (17-19). Many common herbs and spices are claimed to have blood glucose lowering effects that make them useful for the treatment of type 2 diabetes. A large number of pharmacological researches on the antidiabetic effects of medicinal plants resulted in an increase in the number of people who use these natural compounds to control their disease (20,21). Before the discovery of insulin and other blood glucose lowering agents, traditional herbal remedies were used to treat diabetes and related complications. To date, more than 1200 medicinal plants have been shown to possess antidiabetic activities (22,23).

## 2. Objectives

A wide variety of medicinal plants are used in Shiraz to treat various diseases. This study aimed to determine the native medicinal plants recommended by Shirazian herbalists for the treatment of diabetes.

## 3. Materials and Methods

Shiraz is one of the largest cities in Iran and is the capital of Fars province. In 2009, the total population of the city was 1 460 665. Shiraz has a moderate climate and lies in the Zagros mountain range at an altitude of 1468 m. Shiraz city has some famous mountains known as Kuh-e Sabz-Pushan, Kuh-e Bamu, Kuh-e Chel-Magham and Kuh-e Drak. City of Shiraz, with a length of 40 km and a width of 15 to 30 km has a total area of 1268 square kilometers. Shiraz is geographically located in the southwest of Iran and in the central of Fars province. The coldest month of the year is January, with an average temperature of 5°C and the warmest month is July with an average temperature of 30°C. The average annual temperature is about 18°C and the average annual rainfall is 3378 mm.

### 3.1. The methodology of ethno-medicinal data collection

The ethno-medicinal data were collected through direct interview with herbalists and customers between July to September 2015. Herbalists were interviewed in their herbal stores using semi-structured questionnaire and their traditional ethno-medicinal knowledge were recorded. Questionnaires were included herbalist personal information, plant local name, plant growth season, the plant parts used, preparation methods, and traditional therapies. Questionnaires data were transferred to Microsoft Excel.

### 3.2. Ethical issues

1) The research followed the tenets of the Declaration of Helsinki; 2) This project was approved by ethics committee of Medical plants Research Center, Shahrekord University of Medical Sciences, Shahrekord, Iran.

### 3.3. Statistical analysis

The collected data were processed using Microsoft Excel 2007.

## 4. Results

The interview data indicate that 24 species of medicinal plants belonging to 19 families are used for the treatment of diabetes in Shiraz. The families with most antidiabetic species were Compositae (13%), Rosaceae (13%) and Cucurbitaceae (8%). The most frequently used plant parts were fruits (38%) and the most commonly prescribed form was decoction (62%). Ethno- medicinal information of plants used for the treatment of diabetes in Shiraz are shown in Table 1.

**Table 1:** Scientific name, common name, family name, plant parts used, method of application and therapeutic effects of collected plants

**Scientific name**	**Family**	**Persian names**	**Usable Part of plant**	**How to use**	**Traditional use in Shiraz**
*Juglans regia L*	Juglandaceae	Gerdoo	Leaves	Oral	Diabetes
*Cinnamomum verum*	Lauraceae	Darchin	Bark‏	Leaves	Diabetes
*Ficus johannis Boiss.*	Moraceae	Anjir-Vahshi-Daraki	Leaves	Oral	Diabetes
*Lamium amplexicaule L.*	Lamiaceae	Gazaneh Say	Aerial parts	Leaves	Diabetes
*Trigonella monpeliaca L.*	Papilionaceae	Shanbalileh	Aerial parts	Decoction	Diabetes
*Phaseolus vulgaris L*	Leguminosae	Loobia	Aerial parts	Leaves	Diabetes
*Arctium lappa*	Compositae	Baba-adam	Aerial parts	Leaves	Diabetes
*Urtica dioica L*	Urticaceae	Gazaneh	Aerial parts	Leaves	Diabetes
*Olea europaea*	Oleaceae	Zeitoon	Leaves	Oral	Diabetes
*Amygdalus scoparia Spach.*	Rosaceae	Badam-Koohi-Arzhan	Leaves	Leaves	Diabetes
*Salvia officinalis*	Labiatae	Maryam-Goli	Aerial parts	Leaves	Diabetes
*Anethum graveolens dhi*	Apiaceae	Shevid	Leaves	Decoction	Diabetes
*Achillea millefolium L.*	Compositae	Boomadaran-Sefid	Aerial parts	Decoction	Diabetes
*Cotoneaster persica Pojark.*	Rosaceae	Shirkhesht	Aerial parts	Decoction	Diabetes
*Ixillirion tataricum (Pall.) Roem et Schult.*	Amaryllidaceae	Khiarak	Leaves	Decoction	Diabetes
*Securigera securidaca*	Fabaceae	Adas-almolk	Leaves and fruits	Decoction	Diabetes
*Citrullus colocynthis*	Cucurbitaceae	Hendavaneh-Aboojahl	Fruit	Fresh fruit‏ Decoction	Diabetes
*Allium sativum*	Alliaceae	Sir	Balb	Oral	Diabetes
*Lagenaria vulgaris*	Cucurbitaceae	Kedoo	Fruit	Oral	Diabetes
*Curcuma longa*	Zingiberaceae	Zardchooveh	Bark	Leaves	Diabetes
*Gundelia tournefortii*	Compositae	Kangar	Leaves	Oral	Diabetes
*Zataria multiflora*	Lamiceae	Avishan-Shirazi	Leaves	Decoction	Diabetes
*Berberis vulgaris*	Berberidaceae	Zereshk	Fruit	Decoction	Diabetes
*Mespilus germanica*	Rosaceae	Azgil	Fruit	Decoction	Diabetes

The number of mentioned anti-diabetic effects for each plant is shown in Table 2. According to the Table 2, species with the highest frequency of mentions in the interview were *Juglans regia L, Trigonella monspeliaca L., Urtica dioica* and *Amygdalus scoparia Spach.*

**Table 2:** The number of mentions of antidiabetic effects

**Scientific name**	**The No. of herbalists mentioned the plant**	**The total No. of herbalists**	**Frequency of citation (FC) %**
*Juglans regia L*	14	27	51.85
*Cinnamomum verum*	10	27	37.03
*Ficus johannis Boiss.*	3	27	11.11
*Lamium amplexicaule L.*	5	27	18.51
*Trigonella monpeliaca L.*	19	27	70.37
*Phaseolus vulgaris L*	2	27	7.40
*Arctium lappa*	2	27	7.40
*Urtica dioica L*	1	27	40.74
*Olea europaea*	7	27	25.92
*Amygdalus scoparia Spach.*	13	27	48.14
*Salvia officinalis*	4	27	14.81
*Anethum graveolens dhi*	9	27	33.33
*Achillea millefolium L.*	4	27	14.81
*Cotoneaster persica Pojark.*	4	27	14.81
*Ixillirion tataricum (Pall.) Roem et Schult.*	4	27	14.81
*Securigera securidaca*	5	27	18.51
*Citrullus colocynthis*	10	27	37.03
*Allium sativum*	10	27	37.03
*Lagenaria vulgaris*	5	27	18.51
*Curcuma longa*	4	27	14.81
*Gundelia tournefortii*	5	27	33.33
*Zataria multiflora*	4	27	14.81
*Berberis vulgaris*	5	27	18.51
*Mespilus germanica*	5	27	18.51

**Figure 1:**
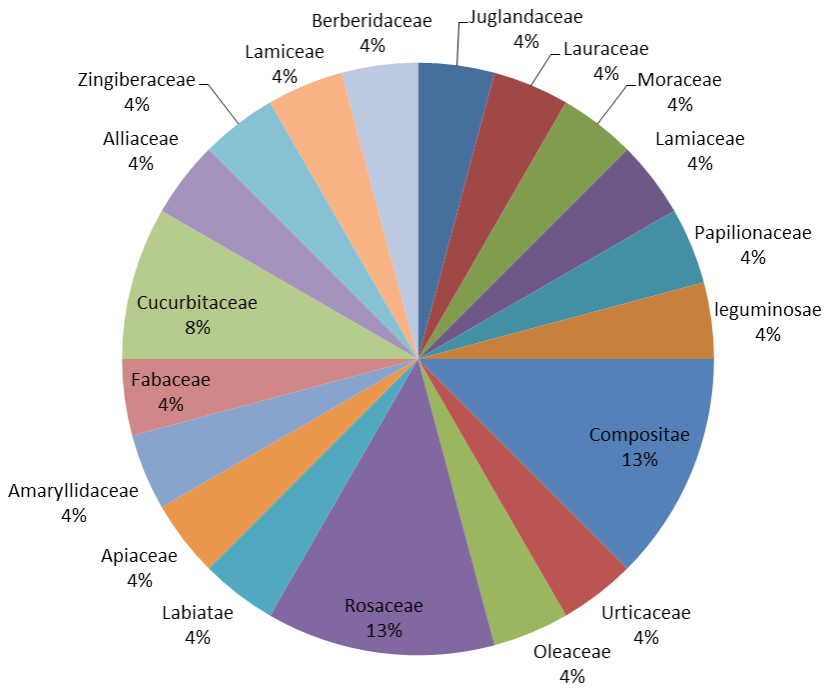
The percentage of botanical family used for the treatment of diabetes in Shiraz

**Figure 2:**
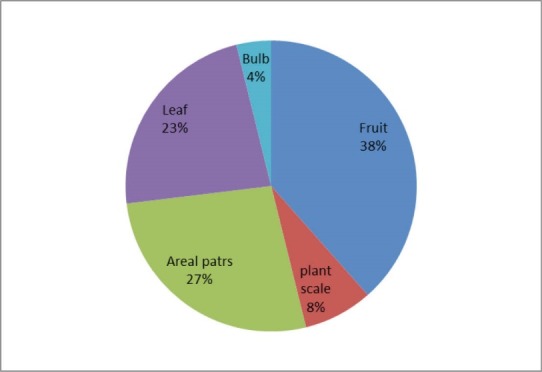
The percentage of plant parts used for the treatment of diabetes in Shiraz.

**Figure 3:**
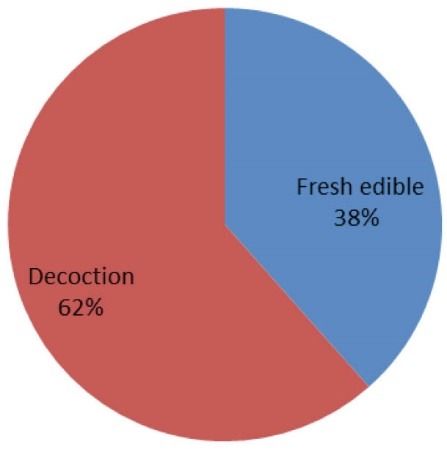
The percentage of applied form of plant for the treatment of diabetes in Shiraz.

The percentage of botanical family used, the used parts and preparation method are shown in Figures 1-3.

## 5. Discussion

Diabetes is the seventh leading cause of death, affecting more than 100 million people each year (5). It has been estimated that the total numbers of patients with diabetes in the world will rise from 150 million in 2003 to 300 million in 2025 (6). The use of medicinal plants is the main component of alternative and complementary medicine (7,8).

A wide variety of medicinal plants are used to treat diabetes in different parts of Iran. In Sistan and Baluchestan province, Nigella (*Nigella sativa L*.) is used to treat diabetes (24). Shaghayegh lubtiz (*Glaucium oxylobum* Boiss &amp; Buhse), shaghayegh goldorosht (*Glaucium grandiflorum Boiss* &amp; *Huet*.), and shengasbi (*Scorzonera cana* (CA Mey) O. Hoffm.) are used to cure diabetes in the Cheshme-Anjir region of Fars province (25). *Teucrium polium* and *Solanum nigrum* are traditionally consumed in Kazerun, Fars province to manage, control, and treat diabetes (26). Bitter melon (*Citrullus colocynthis*), coriander (*Coriandrum sativum*), Barley (*Hordeum vulgare* L.) and esfand (*Peganum harmala*) were reported to be used for the treatment of diabetes in Kerman province (27). In city of Mobarakeh, Isfahan province, walnut (*Juglans regia L*), barley (*Hordeum vulgare L*.), pumpkin (*Cucurbita pepo*), coriander (*Coriandrum sativum L.*) and spearmint (*Mentha spicata L*) are used for this purpose (28). Lorestan people believe that barberry (*Berberis integerrima*), wild pistachio (*Pistacia atlantica*) and summer onions (*Nectaroscordum tripedale*) have antidiabetic effects (29). *Capparis spinosa L*., *Citrullus colocynthis L., Prosopis farcta* are used in Ilam Province to treat diabetes (30). An ethno-medicinal study in Urmia province showed that, *Achillea millefolium L*., *Alyssum desertorum Stapf., Arctium lappa L., Avena sativa L., Berberis integerrima Bunge., Cerasus microcarpa, Cinnamomum verum, Citrullus colocynthis, Coronilla varia L., Crataegus aronia (L.) Bosc ex Dc., Crataegus oxyacantha L., Equisetum arvense L., Juglans regia, L. album L., Nasturtium officinalis (L.) R. Br., Nepeta bracteata Benth., Nepeta meyeri Benth., Papaver rhoeas L., Polygonum aviculare L., Rhus coriaria L., Rosa foetida Hermam., Rumex sculantus L., Salvia nemorosa L., Sanguisorba minor Scop., Sophora alopecuroides, Teucrium orientale L., T. polium L., Trifolium pratense L., Trifolium purpureum Loisel., and Urtica dioica L*. are commonly recommended by herbalists to treat diabetes (31).

This study reviewed the native medicinal plants recommended by Shirazian herbalists for the treatment of diabetes. The results showed that, a total number of 24 plants are traditionally used to treat diabetes in Shiraz. These plants include, *Juglans regia L, Cinnamomum verum, Ficus johannis Boiss, Lamium amplexicaule L., Trigonella monspeliaca L., Phaseolus vulgaris L., Arctium lappa, Urtica dioica L., Olea europaea, Amygdalus scoparia Spach., Salvia officinalis., Anethum graveolens dhi, Achillea millefolium L., Cotoneaster persica pojark., Ixillirion tataricum (Pall.) Roem et Schult., Securigera securidaca., Citrullus colocynthis., Allium sativum., Lagenaria vulgaris., Curcuma longa., Gundelia tournefortii., Zataria multiflora., Berberis vulgaris, and Mespilus germanica.* Thirty-three percent of recommended medicinal plants in this study were also mentioned by Urmia herbalists.

The antidiabetic effects of some of these plant species have been investigated in animal and humane models of diabetes. Species with considerable antidiabetic effects are *J. regia*, *Cinnamomum verum*, *Arctium lappa*, *Olea europaea*, *Amygdalus scoparia*, *Anethum graveolen*, *Securigera securidaca*, *Citrullus colocynthis*, *Allium sativum* and *Zataria multiflora* (32-35). The antidiabetic effects of 44% of recommended medicinal plants in this study have been demonstrated in experimental studies. Interestingly, most of these plants have therapeutic effects on diabetic associated diseases, too. More studies are needed to assess the antidiabetic effects of the recommended plants by Shirazian herbalists. Their effective compounds and mechanism of action are also suggested to be determined. In the case of effectiveness, these plants can be processed and refined to produce drugs

The mechanisms by which these plants lower blood glucose are not clear. Although, it has been shown that various herbal components such as phenolics, tannins, saponins and alkaloids have antidiabetic properties, flavonoids and phenolic components are characterized as the main blood glucose lowering components (32-35). If we accept this hypothesis, thus, other medicinal plants with phenolic and antioxidant compounds may possess anti diabetic property. Furthermore, considering the importance of these plants in reducing blood glucose, more investigations are recommended to scientifically confirm the effects of these plants. Following the confirmation of the effects, more researches are also needed to analyze the effective compounds and to introduce new hypoglycemic drugs.

## 6. Conclusions

This study introduced important plants species which were recommended by local herbalist of Shiraz for the treatment of diabetes. A total number of 24 plants are recommended for the treatment of diabetes. For 45% of reported plants, pharmaceutical studies approved antidiabetic effects in animal or humane model of diabetes. The most frequently used part was fruits and the most commonly used preparation methods was decoction.

## Acknowledgments

Authors thank to all peoples who helped us in this study.

## Authors’ contribution

All the authors wrote the first draft of the manuscript equally. MRK revised and edited the final draft.

## Conflicts of interest

The authors declared no competing interests.

## Funding/Support

The authors disclosed receipt of the following financial support for the research, authorship, and/or publication of this article: This article was prepared by support of Research Deputy of Shahrekord University of Medical Sciences (Grant# 8437996).
